# Scalp Cooling as a Biopsychosocial Intervention in Patients Receiving Highly Alopecia-Inducing Chemotherapy: Prospective Single-Arm Study

**DOI:** 10.2196/94635

**Published:** 2026-07-20

**Authors:** Dörthe Schaffrin-Nabe, Anke Josten-Nabe, Adrian Heinze, Andrea Tannapfel, Merle Schaffrin, Rudolf Voigtmann

**Affiliations:** 1 Praxis für Hämatologie und Onkologie, Universitätsstr. 110 e, Bochum, 44799, Germany, 49 17647937288; 2 Independent Research Consultant, Hamburg, NRW, Germany; 3 University Medical Center Hamburg-Eppendorf, Hamburg, Germany; 4 Bürkle-de-la-Camp-Platz 1, Pathologie Ruhr-Universität Bochum, 44789, NRW, Germany

**Keywords:** scalp cooling, chemotherapy-induced alopecia, hair follicle biology, ultrastructural analysis, quality of life, cognitive appraisal, supportive oncology, patient-reported outcomes

## Abstract

**Background:**

Chemotherapy-induced alopecia is among the most psychologically distressing adverse effects of systemic cancer therapy. Although scalp cooling is increasingly used to mitigate hair loss, it is still largely perceived as a cosmetic intervention. Its broader psychological relevance and the biological basis of treatment success, particularly the preservation of follicular integrity under ongoing cytotoxic exposure, remain insufficiently explored.

**Objective:**

This study aimed to reconceptualize scalp cooling beyond visible hair preservation by examining its psychological impact on patients receiving highly alopecia-inducing chemotherapy, while integrating quantitative objective hair preservation metrics with structural and ultrastructural analyses of hair follicle damage to identify avenues for improving follicular integrity and scalp-cooling efficiency.

**Methods:**

A total of 82 patients undergoing highly alopecia-inducing chemotherapy consisting of a sequential anthracycline-taxane regimen (4 cycles of epirubicin and cyclophosphamide followed by 12 weekly paclitaxel applications) received standardized scalp cooling. Objective hair preservation was quantified using the hair mass index (HMI) as a standardized and reproducible measure of hair retention. Structural and ultrastructural follicular integrity was assessed using light microscopy as well as scanning and transmission electron microscopy. Objective hair preservation metrics were analyzed in relation to patient-reported quality-of-life outcomes (EORTC [European Organisation for Research and Treatment of Cancer]–based measures), subjective treatment burden, and cognitive appraisal of the scalp-cooling experience. Multivariable regression models were applied to identify determinants of posttherapeutic quality of life.

**Results:**

Visible chemotherapy-induced alopecia was successfully prevented in more than half of the treated patients. Scalp cooling resulted in substantial objective hair preservation as quantified by the HMI. However, HMI values showed only a limited association with posttherapeutic quality-of-life outcomes. In contrast, the cognitive appraisal of scalp cooling emerged as a central determinant of posttherapeutic quality of life, independent of the degree of objective hair retention. Structural and ultrastructural analyses demonstrated that the preservation of follicular integrity was closely associated with successful macroscopic hair retention under ongoing cytotoxic exposure, supporting a biological basis for the clinical effectiveness of scalp cooling.

**Conclusions:**

The clinical relevance of scalp cooling extends beyond objective and visible hair preservation and appears to reside predominantly in its psychological impact on patients undergoing highly alopecia-inducing chemotherapy. Importantly, the identification of structural and ultrastructural markers of follicular vulnerability provides a mechanistic foundation for the future optimization of scalp-cooling approaches and for the development of adjunct follicle-directed protective strategies to enhance follicular integrity and support patient well-being during cytotoxic therapy.

## Introduction

### Background

Chemotherapy-induced alopecia (CIA) remains one of the most visible and psychologically distressing adverse effects of systemic cancer therapy. Beyond a transient cosmetic change, hair loss functions as a public marker of disease, disrupts personal identity, and continuously reinforces the illness experience for patients and their families [1,2]. Despite substantial advances in oncologic treatment, the psychosocial burden of CIA remains inadequately addressed.

Scalp cooling is currently the only US Food and Drug Administration–approved intervention shown to mitigate CIA and has gained broad clinical acceptance. However, its efficacy is highly heterogeneous: while some patients achieve near-complete hair preservation, others experience pronounced alopecia despite identical chemotherapy regimens and standardized cooling protocols [3,4]. This interindividual variability represents a major limitation of current approaches and indicates that key determinants of treatment response remain insufficiently understood.

Although direct clinical evidence remains limited, experimental human hair-follicle models and scalp-cooling outcome studies support the biological plausibility that intrinsic follicular characteristics, including hair-shaft integrity and pigmentary-unit stability, may contribute to interindividual variation in chemotherapy-induced damage and scalp-cooling response [5-10]. At the same time, the subjective experience of hair loss and individual coping strategies substantially shape quality of life, highlighting CIA as an interface between biological vulnerability and psychosocial resilience.

In this context, this study examines scalp-cooling efficacy in a homogeneously treated cohort receiving highly alopecia-inducing chemotherapy. By integrating objective follicular parameters with patient-reported outcomes (PROs), this work aims to contribute to a more nuanced, biopsychosocial understanding of hair preservation and to enable a more individualized approach to supportive care strategies [6,11].

### Study Population

The study enrolled 82 patients with early-stage breast cancer, all of whom received curative-intent chemotherapy. A uniform treatment regimen was applied, consisting of epirubicin and cyclophosphamide followed by weekly paclitaxel. This standardized approach ensured comparable cytobiological effects on hair follicles across the cohort and provided a consistent basis for evaluating scalp-cooling efficacy. Given the predictable and uniform severity of alopecia associated with this regimen, a control group without scalp cooling was not included.

Scalp cooling was performed with a sensor-controlled device designed to maintain a consistent scalp temperature below 22 °C. By ensuring reproducibility in both chemotherapy and cooling parameters, the study sought to isolate patient-specific and follicle-specific determinants of hair preservation. Patients were excluded from scalp cooling in cases of clinically manifest scalp metastases, generalized hematologic malignancies such as leukemia, scalp injuries, or cryoglobulinemia.

### Objectives

The primary objective of the study was to evaluate the efficacy of scalp cooling for hair preservation during chemotherapy, using the hair mass index (HMI) as an objective measure obtained through cross-sectional trichometry.

Three primary efficacy end points were defined:

1. Posttreatment HMI as a measure of residual hair mass,
2. Avoidance of visible alopecia, defined as HMI ≥50,
3. Absolute hair loss, calculated as the difference between posttreatment and pretreatment HMI (ΔHMI).

Secondary objectives included the characterization of treatment-associated trichological and ultrastructural hair alterations, the exploratory assessment of biological and clinical factors associated with hair preservation, and the evaluation of patient-reported treatment experiences and the psychosocial burden related to hair loss and scalp cooling. Trichological and biological secondary outcomes included pretreatment and posttreatment assessments of hair density, anagen rate, and hair shaft and bulb diameter, as well as ultrastructural hair-shaft surface alterations. Particular emphasis was placed on melanosomal features, with transmission electron microscopy (TEM) used to quantify melanosome morphology and density.

Potential predictors and influencing factors of hair preservation were evaluated across multiple domains, including general health parameters (eg, age, comorbidities, alopecia-inducing co-medications, nicotine use, and menopausal status), individual hair characteristics and practices (eg, hair color, length, structure, and hair treatment), and treatment-related biological vulnerability markers. These included hematologic toxicity during chemotherapy (eg, neutrophil nadirs) as well as laboratory parameters reflecting liver function, renal function, and thyroid status. In female patients, endocrine profiling (follicle-stimulating hormone, luteinizing hormone, and estradiol) was explored to contextualize gonadal function.

PROs were collected to assess the subjective experience of scalp cooling, including perceived burden and treatment acceptance. Psychosocial impact was evaluated before and after the intervention using selected items derived from the EORTC QLQ-C30 (European Organisation for Research and Treatment of Cancer Quality of Life Questionnaire), complemented by study-specific questions to capture treatment-related psychosocial effects associated with hair loss and the cooling procedure.

## Methods

### Overview

To determine hair density, measurements were carried out at the frontal vertex before and after treatment using the Cross Section Trichometer (Cohen HMI device) [12]. This tool enables standardized quantification of hair mass within a 4-cm² scalp area. The procedure was designed to deliver consistent, reproducible results at single time points, assuming the examiner had received appropriate training. Only hairs longer than 2.5 cm were considered. Outcomes were expressed as the HMI, defined as the surface area of hair in mm² per cm² of scalp, multiplied by 100. Values typically fall between 75 and 100; a score ≥50 was predefined as the threshold for the absence of clinically visible alopecia consistent with established HMI methodology, indicating that visible thinning typically emerges below this level [12], while values below 20 correspond to advanced alopecia [12]. For analysis, around 50 follicles were extracted from the selected 4 cm² scalp region and positioned between 2 glass slides with the roots exposed, sampling for reproducible trichogram assessment. At the same time, the number of extracted follicles was intentionally kept as low as possible to minimize additional hair removal in a psychologically vulnerable patient population, with the explicit aim of hair preservation [12].

Light microscopy was applied to capture images of untreated hair samples at magnifications of 10× and 40× using a Leica DM3000 (Leica Microsystems) microscope. Measurements included bulb diameter (typical range: 150‐200 µm) and shaft thickness (normal for the frontal vertex: 75‐85 µm) [13]. The growth phase of the follicles was assessed based on the characteristic bulb morphology: according to established trichogram and hair cycle morphology criteria, anagen follicles showed normal bulb dimensions, while catagen and telogen follicles presented with smaller bulbs and club-shaped ends. Structural abnormalities such as shaft irregularities, breakage, and deformation were interpreted as signs of follicular dystrophy [14].

**Figure 1.**
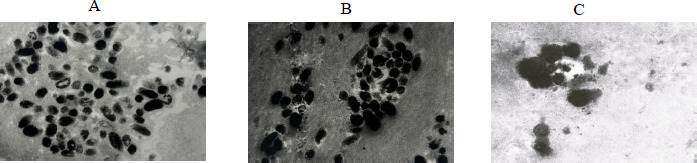
Exemplary transmission electron microscopy photos. (A) Patient with brown dense hair (pre-HMI=100). Pretherapeutic pigmentary status: 89 total melanosomes, 44 pheomelanosomes, 45 eumelanosomes, and ratio of eumelanosomes to pheomelanosomes=1.02. Scale: approximately 8000:1. (B) Same patient with no visible hair loss (post-HMI=58). Posttherapeutic pigmentary status: 75 total melanosomes, 31 pheomelanosomes, 44 eumelanosomes, and ratio of eumelanosomes to pheomelanosomes=1.42. Scale: approximately 8000:1. (C) Patient with bad hair retention beginning of visible alopecia (post-HMI=35). Pigmentary status: disordered melanogenesis characterized by clumping of melanin. Scale: approximately 12,000:1. HMI: hair mass index.

To study melanosome distribution, TEM was applied to ultrathin hair shaft sections obtained from 52 participants before and after treatment (Figure 1). These cases represented the first consecutively available samples for which electron microscopic processing was feasible. The subset was not selected according to clinical outcomes, degree of alopecia, treatment response, or any other biological characteristics. Rather, the number of evaluable cases was determined by the substantial technical complexity, limited pathology resources, and the labor-intensive nature of ultrastructural tissue preparation, image acquisition, and quantitative analysis. Consequently, no intentional enrichment for responders, nonresponders, or specific morphological patterns was performed, minimizing the risk of selection bias. Two types of organelles were identified: pheomelanosomes, typically smaller and more rounded, and eumelanosomes, which tend to be elongated and fibrillar in form. The quantitative assessment showed that blond hair contained relatively few melanosomes (about 9 per 100 µm²), while markedly higher densities were observed in darker hair, averaging around 66 per 100 µm² in brown and 70 per 100 µm² in black samples [15]. For preparation, follicles were fixed in glutaraldehyde, dehydrated through successive steps, and embedded in Epon. Sections approximately 50-nm thick were cut, stained with uranyl acetate and lead citrate, and examined at magnifications ranging between 1500× and 15,000× (JEOL 2100).

**Figure 2.**
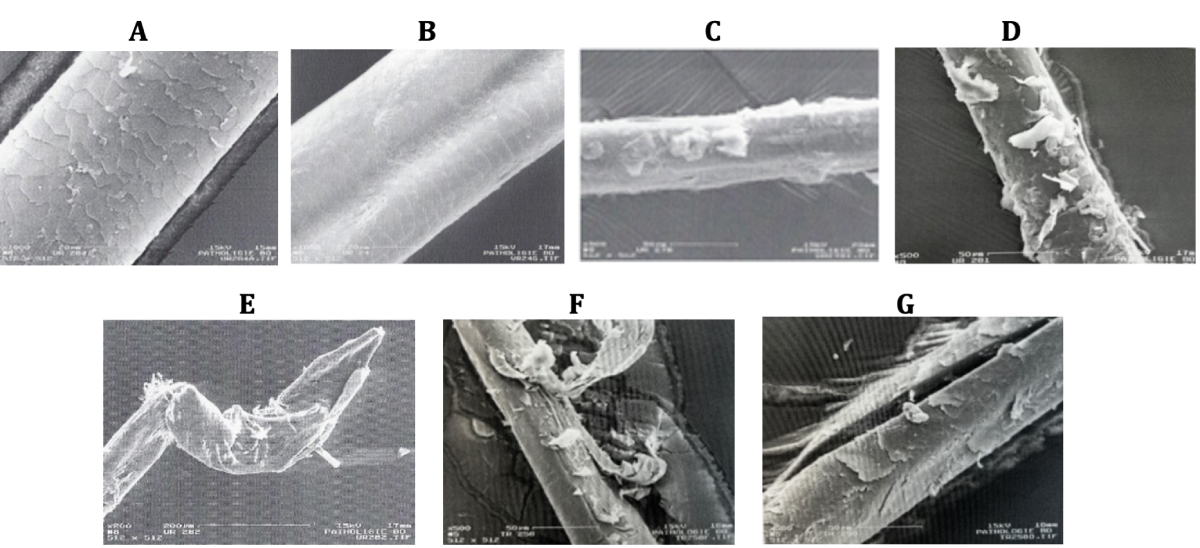
Different patterns and grades of hair shaft damage. (A) Normal roof tile scale pattern; (B) Dents, grade 1; (C) Deposits on hair shaft, grade 1; (D) Lift-ups of cuticle, grade 2; (E) Distortion and crack of hair shaft, grade 3; (F) Severe lift-ups with partial exposure of cortex; (G) Extensive exposure of cortex with dents, deposits, and cracks, grade 4.

Scanning electron microscopy (SEM) was applied to evaluate surface alterations of the hair shaft in untreated and treated samples. Examinations were performed with a Zeiss Gemini 982 (ZEISS Microscopy) field emission SEM at magnifications ranging from 200× to 2000×. Prior to imaging, the hair was sputter-coated with a thin gold layer. The extent of structural impairment was categorized using a system comparable to the Kim scale, which is commonly used for classifying extrinsically induced hair damage. For illustration, representative patient samples showing different levels of hair surface deterioration are displayed in Figure 2 [16].

The Kim scale (adapted) is as follows:

- Grade 0: intact, untreated hair with a uniform cuticle layer.
- Grade 1: slight irregularities of the cuticle, including shallow dents or uneven overlap, without cracks or loss of material.
- Grade 2: pronounced lifting of cuticle layers, with fissures or small perforations, but the cortex is still covered.
- Grade 3: partial exposure of the underlying cortex.
- Grade 4: complete loss of the cuticle layer, leaving the cortex unprotected.

Sections of hair shafts from 52 participants were examined to confirm consistency in the detected alterations. The analysis concentrated on grading intrinsic surface damage from 0 to 4 in relation to the therapeutic procedure.

### Patient-Centered Outcomes (Self-Report Measures)

Patient-centered outcomes were assessed using self-report measures obtained at baseline and after the intervention. The subjective appraisal of treatment and the overall quality of life were rated on Likert-type scales ranging from 1 (very poor) to 7 (very good). Treatment-related burden and discomfort were assessed on a 4-point scale from 1 (none) to 4 (very strong).

Quality-of-life domains were evaluated using selected items from the EORTC QLQ-C30, complemented by additional study-specific questions addressing treatment experience and procedure-related aspects.

Patient-reported questionnaires were used to capture patients’ subjective treatment experiences, perceived burden, and overall well-being. Psychosocial data were collected prospectively from the study’s initiation onward as part of the observational study protocol. Consequently, analyses examining associations between cognitive appraisal, hair preservation, and quality of life were conducted using prospectively collected data.

### Statistical Procedures

Descriptive statistics (median, IQR, and percentage) were used to summarize patient and treatment characteristics, as well as hair retention outcomes. Multiple linear and binary-logistic regression models were conducted as exploratory analyses to assess associations with hair retention and quality of life. Because some variables included in the models were measured after treatment, the models were not intended to strictly establish causal or temporal effects. Baseline variables were interpreted as potential predictors, whereas posttreatment parameters were interpreted as correlates of the corresponding outcome. The joint approach was used to examine these associations while simultaneously adjusting for baseline characteristics and posttreatment morphological parameters.

All key model assumptions were assessed and met for all models, including linearity, normality, and homoscedasticity of residuals, absence of influential outliers, and acceptable multicollinearity. For the 3 outcome variables of hair retention (post-HMI, HMI≥50, and HMI difference), clinically relevant and biologically plausible correlates were identified within thematic domains and entered into multivariable models if they were at least borderline significant (*P*=.006). Only standardized regression coefficients are reported, and the intercept was omitted from the models’ tables because it is not interpretable in standardized form. Finally, we used Spearman correlation coefficients to test associations between melanosomes and damage degree because of their ordinal scale and potential outliers. Statistical significance was defined as *P*=.003.

### Ethical Considerations

This study was approved by the Research Ethics Committee (2016‐011-f-S Ethikkommission Westfalen-Lippe, Münster, Germany). All research data were deidentified and coded with unique identifiers, and the data were transmitted using secure encrypted protocols and stored on encrypted disks on secure servers. The study included only patients who had agreed to the use of their medical information by signing the respective consent form. No compensation was provided to participants for survey completion. This paper does not include any images or information that could identify individual participants.

## Results

Among 82 patients, 80 (97.6%) were women, and the median age was 56.5 (IQR 49‐65) years. The baseline median HMI score was 75 (IQR 58‐92) and declined after treatment to a median of 51 (IQR 17‐58). At follow-up, 52% (n=43) of participants still had an HMI score of 50 or higher, corresponding to the absence of visible hair loss. For patients with initially low hair density, maintaining the HMI may be of particular clinical importance, highlighting the role of relative HMI preservation as a meaningful end point. The median decline in the HMI score from baseline to posttreatment was −27 (IQR −45 to −10). In those starting with an HMI below 50 (n=12), the median reduction amounted to −19 (IQR −27 to −5). Comprehensive baseline characteristics, treatment-related factors, and posttreatment clinical parameters together with variables derived from light microscopy, TEM, and SEM are summarized in Table S1 in Multimedia Appendix 1. Complete ultrastructural data were available for a quality-controlled subset of 52 patients.

While longitudinal changes in HMI provided a robust quantitative measure of treatment-associated alterations in scalp hair density, this global metric does not fully capture the underlying biological mechanisms at the follicular level. To contextualize these numerical changes, we also examined the microstructural integrity of individual hair follicles using complementary morphological approaches. These microscopic assessments capture complementary aspects of follicular and hair shaft integrity, encompassing surface morphology—particularly ultrastructural damage features—as well as pigmentation patterns, which cannot be adequately reflected by global quantitative indices alone.

In order to comprehensively characterize hair retention outcomes, 3 complementary HMI–based efficiency parameters were evaluated: (1) posttreatment HMI as a measure of residual scalp hair density, (2) the proportion of patients maintaining an HMI ≥50, corresponding to the absence of visible hair loss, and (3) the absolute change in HMI from baseline, capturing individual longitudinal dynamics and relative hair preservation. Together, these parameters allow for a differentiated assessment of treatment-associated effects on scalp hair beyond absolute posttreatment values.

The initial modeling steps are summarized in Tables S2-S5 in Multimedia Appendix 1, showing multivariable regression analyses thematically grouped by general clinical characteristics, as well as light, TEM, and SEM variables to identify independent factors associated with each of the 3 HMI efficiency parameters.

These supplementary tables provide the structural and biological context for the subsequent efficiency-focused analyses and are presented separately to maintain clarity and readability of the main results. Only variables meeting the predefined relevance threshold of *P=*.08 were retained in the final multivariable models for posttreatment HMI, HMI ≥50, and absolute HMI change and are therefore presented in Tables1^3^.

**Table 1.** Final exploratory linear regression for associations with posttreatment hair mass index^a^.

Variables	β (95% CI)	*P* value
Comorbidity	−.28 (−0.58 to 0.02)	.07
Neutrophil	.17 (−0.12 to 0.46)	.25
Anagen post	.3 (−0.01 to 0.61)	.06
Pheomelanosome difference	.17 (−0.12 to 0.46)	.25
Damage difference	−.29 (−0.58 to -0.01)	.046

a Model fit was adjusted *R*²=0.32.

**Table 2.** Final exploratory binary-logistic regression for associations with hair mass index ≥50^a^.

Influencing variables	Odds ratio (95% CI)	*P* value
Comorbidity	0.05 (0.01‐0.75)	.05
Neutrophil	2.46 (0.60‐25.69)	.32
Hair shaft pre	1.12 (1.04‐1.27)	.02
Bulb size pre	0.98 (0.95‐1.01)	.26
Bulb size post	1.02 (0.98‐1.06)	.39
Damage difference	0.28 (0.06‐1.06)	.08

a Model fit was Nagelkerke *R*²=0.65.

**Table 3.** Final exploratory linear regression for associations with the hair mass index difference^a^.

Variables	β (95% CI)	*P* value
Age	.31 (0.05 to 0.58)	.02
Medication	−.35 (−0.61 to −0.09)	.009
Bulb size pre	.05 (−0.21 to 0.31)	.69
Pheomelanosome difference	.34 (0.09 to 0.59)	.009
Eumelanosome difference	.27 (0.01 to 0.53)	.04

a Model fit was adjusted *R*²=0.33.

To address the temporal structure of the data, baseline variables and posttreatment variables were conceptually distinguished. Baseline variables were analyzed as potential predictors of treatment outcomes, whereas posttreatment variables were included to characterize biological features associated with treatment responses. Accordingly, the models including posttreatment variables should be interpreted as exploratory analyses of associations rather than predictive models in a strict temporal sense.

When analyzing factors associated with posttreatment HMI (Table 1), the degree of hair shaft damage difference showed a statistically significant negative association (β=−.29, *P*=.046), indicating that greater increases in hair shaft damage were linked to lower posttreatment HMI values. Comorbidity exhibited a borderline negative association (β=−.28, *P*=.066), suggesting a tendency toward reduced hair retention in patients with comorbid conditions. Posttreatment anagen frequency showed a borderline positive association (β=.30, *P*=.06), indicating a trend toward improved posttreatment HMI with higher anagen proportions.

For HMI ≥50 (Table 2), the baseline hair shaft diameter was significantly associated with visible hair retention (odds ratio [OR] 1.12, 95% CI 1.04-1.27; *P*=.02), indicating that a larger baseline hair shaft diameter was linked to higher odds of maintaining visible hair. Comorbidity showed a borderline negative correlation (OR 0.05, 95% CI 0.01-0.75; *P*=.05), suggesting a tendency toward a reduced likelihood of HMI-defined hair preservation (HMI≥50). A greater increase in hair shaft damage was also related to lower odds of preventing visible hair loss, although this did not reach statistical significance (OR 0.28, 95% CI 0.06-1.06; *P*=.08). No relevant associations were observed for neutrophil count or bulb size parameters. Overall, model performance was very good (Nagelkerke *R*²=0.65).

Finally, in the linear model for HMI difference (Table 3), age (β=.31, *P*=.02), pheomelanosome difference (β=.34, *P*=.009), and eumelanosome difference (β=.27, *P*=.04) were significantly associated with the HMI change, indicating that older age and lower degrees of melanosome loss were linked to a smaller decline in the HMI. Medication use showed a significant inverse relationship to greater HMI reduction among patients receiving alopecia-inducing medication (β=−.35, *P*=.009). Overall, the model fit was moderate (adjusted *R*²=0.33).

Given the exploratory design and the number of tested influencing factors across models, no formal adjustment for multiple testing was applied; the results should therefore be interpreted as exploratory. CIs were wide for some estimates, reflecting the limited precision associated with the sample size.

In addition to biological and treatment-related variables, psychosocial parameters were specifically examined for their association with hair retention outcomes. This analysis, summarized in Table 4, addresses the psychological dimension of hair preservation and its contribution to the overall treatment experience and quality of life. Finally, in a linear regression model using posttherapeutic quality of life as the dependent variable, we entered pretreatment quality of life, subjective appraisal of scalp cooling, emotional burden, and HMI difference as candidate variables. QoL pre (β=.41, *P*<.001) and scalp-cooling appraisal (β=.53, *P*<.001) showed significant positive associations with moderate-to-strong effect sizes, indicating that higher baseline QoL and a more favorable appraisal of scalp-cooling outcomes were linked to better QoL after therapy. Age, burden, and HMI change were not independently associated with posttreatment quality of life. Overall, the model fit was high (adjusted *R*²=0.48).

**Table 4.** Final exploratory linear regression for associations with posttreatment quality of life (QoL)^a^.

Variables	β (95% CI)	*P* value
Age	.04 (−0.17 to 0.26)	.69
QoL pre	.41 (0.21 to 0.62)	<.001
Appraisal	.53 (0.27 to 0.79)	<.001
Burden	.05 (−0.19 to 0.29)	.67
HMI^b^ difference	−.01 (−0.24 to 0.23)	.97

a Model fit was adjusted *R*²=0.48.

b HMI: hair mass index.

## Discussion

### Principal Findings

This study provides an integrated analysis of scalp cooling that extends beyond the traditional dichotomy of hair preservation versus alopecia. In a cohort exposed to highly alopecia-inducing chemotherapy, visible hair loss was avoided in 53% (n=43) of patients, representing a clinically meaningful level of effectiveness that exceeds purely cosmetic expectations. Importantly, this efficacy signal was consistently accompanied by distinct structural and ultrastructural features of follicular integrity, indicating that treatment response is biologically modulated rather than binary. These findings further suggest that the effectiveness of scalp cooling is not solely determined by temperature reduction per se but reflects a more complex biological response at the level of follicular resilience. While these observations are biologically plausible, they should be interpreted as exploratory. This study was not designed to establish causal mechanistic relationships but rather to identify clinically relevant associations. In particular, relative melanosome stability and hair shaft surface integrity appear to represent a coherent biomarker constellation of follicular health, supporting the concept that scalp cooling modulates intrinsic follicular vulnerability to chemotherapy-induced damage rather than acting as a purely physical cooling intervention. However, these structural features were assessed in a limited subset of samples using high-resolution ultrastructural techniques, reflecting their technically demanding nature and should therefore be interpreted with caution.

Previous large-scale registry data, such as the Dutch Scalp Cooling Registry, including more than 7400 patients, have consistently demonstrated that the type of chemotherapy regimen is a key determinant of scalp-cooling efficacy [17]. Consistent with these findings, we have similarly observed variability in scalp-cooling efficacy across different treatment settings in previous work [18]. In addition, we have reported variable success rates across different regimens in prior work. In contrast, this study applied a uniform chemotherapy protocol consisting of epirubicin and cyclophosphamide followed by weekly paclitaxel, a regimen invariably associated with complete alopecia in the absence of scalp cooling. This approach was deliberately chosen to minimize treatment-related heterogeneity and to allow for the assessment of scalp-cooling effects within a highly standardized clinical setting. However, the absence of a control group represents a methodological limitation, as comparative conclusions regarding absolute treatment effects cannot be drawn. At the same time, the use of a uniform chemotherapy regimen allowed for a more focused analysis of interindividual variability in follicular response under comparable treatment conditions. Furthermore, establishing an untreated control group was considered ethically challenging, given the expected near-complete alopecia associated with this regimen.

Although prior investigations have primarily focused on the chemotherapy regimen type and general patient-related factors, our analysis extends this perspective by systematically addressing structural and ultrastructural determinants of follicular preservation.

Our findings indicate that the relative preservation of melanosome abundance—particularly pheomelanosomes—together with a larger pretreatment hair shaft diameter and minimal posttherapeutic shaft surface damage, represents independent correlates of successful hair preservation.

Importantly, these observations should be interpreted as biologically plausible associations rather than strictly causal relationships. Nevertheless, they consistently point toward a central role of melanogenesis integrity and keratinocyte proliferative capacity in maintaining follicular resilience under cytotoxic stress. In this context, chemotherapy-induced impairment of melanogenesis and keratinocyte turnover appears to directly compromise follicular homeostasis. While scalp cooling may attenuate these effects, it does not fully prevent them, thereby highlighting both the biological potential and the inherent limitations of the intervention.

Collectively, these findings support the concept that scalp-cooling efficacy is not solely determined by physical temperature modulation, but rather reflects a biologically modulated preservation of follicular integrity.

From a pathophysiological perspective, anthracyclines and taxanes preferentially target rapidly proliferating keratinocytes within the hair matrix while simultaneously inducing the formation of reactive oxygen species (ROS). This dual mechanism not only disrupts keratinocyte turnover but also functionally impairs components of the follicular pigmentary unit, including melanocytes and associated sebocytic structures [19-21].

Even in the presence of scalp cooling, the residual exposure of follicular melanocytes to cytotoxic agents may induce a prematurely dysregulated state of melanogenesis. Morphologically, this may manifest as increased melanin aggregation, reduced melanosome density, and a compositional shift from pheomelanin toward eumelanin synthesis [19,22].

This shift is mechanistically plausible in the context of oxidative stress, as cysteine—a critical substrate for pheomelanin synthesis—is increasingly diverted toward glutathione production under conditions of elevated ROS. Consequently, the balance of melanogenic pathways is altered in favor of eumelanogenesis, thereby reflecting an adaptive but functionally disruptive antioxidative response [23-25].

These mechanisms provide a molecular basis for the observed reduction in melanosome density and the elevated eumelanin-to-pheomelanin ratio post treatment (Mdn_pre_=0.34 to Mdn_post_=0.52; Table S1 in Multimedia Appendix 1). Importantly, the role of melanosomes extends far beyond the mere determination of hair color. Their perinuclear distribution around keratinocyte nuclei confers both photoprotective and antioxidative benefits, safeguarding keratinocyte genomic stability and ensuring controlled proliferative activities that ultimately drive proper hair shaft development [26-28].

**Figure 3.**
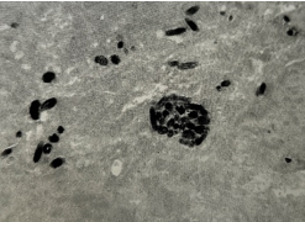
Cluster (more than 5 touching pigments).

Posttherapeutically, occasional melanosome clustering (Figure 3) was observed. However, this feature did not show a significant association with hair retention, suggesting that clustering represents a secondary or epiphenomenal structural alteration rather than a determinant of follicular resilience.

In contrast, preserved melanosome abundance and the relative stability of melanosomal organization emerged as a potential surrogate marker associated with more favorable hair preservation. Minor qualitative shifts in melanosome composition were observed but were not formally quantified and are therefore reported descriptively. These exploratory and hypothesis-generating observations may reflect lower treatment-associated cellular stress and could represent a morphological correlate of relative follicular resilience. Preservation of melanosomal architecture thus extends beyond cosmetic relevance and may contribute to the maintenance of hair shaft integrity. Melanocytes and keratinocytes exist within a tightly coordinated functional unit characterized by reciprocal paracrine signaling and direct cellular cross talk. Mediators such as stem cell factors, endothelin-1, and basic fibroblast growth factor contribute to maintaining melanogenic activity and broader follicular homeostasis. This coordinated interaction provides a biological framework, linking the preservation of melanosomal architecture with follicular stability, and may help explain why the maintenance of an intact melanogenic process is associated with improved hair preservation [26,29]. Although both melanosome stability and shaft-surface integrity contribute significantly to hair retention under scalp cooling, statistical modeling identified them as independent correlates. This phenomenon can be explained by their distinct biological roles: the relative preservation of melanosome density provides intracellular photoprotection and antioxidative buffering, whereas shaft-surface conditions primarily determine the mechanical resilience of the emergent hair fiber. Therefore, each factor contributes additively, but not redundantly, to follicular protection, highlighting the multifactorial nature of CIA [27,28,30].

Increased oxidative cellular stress resulting from residual exposure to cytostatic agents can compromise both the hair bulb and the hair shaft surface. In contrast to metabolically active cells, the extracutaneous compartments of the hair shaft lack sophisticated antioxidative defense mechanisms, including enzymatic and nonenzymatic radical scavenging systems. As a consequence, structural components of the hair shaft remain particularly vulnerable to oxidative degradation, thereby amplifying the impact of chemotherapy-induced injury [31-33].

ROS compromise the hair’s protective lipid envelope, most notably the 18-methyleicosanoic acid monolayer, which is crucial for shaft cohesion. Loss of this barrier renders the cuticle highly susceptible to endogenous and environmental damage. Under chemotherapy, excessive ROS not only damage cuticular elements but also affect cortical structures, leading to fibrillary misalignment. Subsequent oxidative events, including keratin modification, disruption of disulfide cross-links, and alteration of amino acid residues, irreversibly deteriorate the cortical architecture, resulting in fragile, dull, and fracture-prone hair fibers [32,34-37].

From a clinical perspective, the significance of hair shaft surface damage cannot be overstated. SEM demonstrated that a higher damage grade is associated with visible alopecia or lower HMI in general, and descriptive results appear to suggest that a damage grade of 3 (cortical exposure) may constitute a critical threshold for visible alopecia (Table S6 in Multimedia Appendix 1). This observation parallels earlier findings in cosmetic dermatology, where chemical cuticle disruption progressing to cortical exposure invariably results in irreversible hair fragility [16,37].

Within the follicular pigmentary unit, sebocytes act as integral partners to keratinocytes and melanocytes. Their secretory activity not only supports hair shaft emergence but also contributes to the antioxidative and immunomodulatory milieu of the follicle. Anthracycline-based chemotherapy may impair the sebocyte function, thereby reducing the protective capacity of sebum and increasing hair shaft vulnerability [14,19,38,39].

The lipid-rich sebum film reduces friction, minimizes breakage, and provides a hydrophobic barrier that limits transepidermal water loss, chemical irritation, and microbial penetration [40]. Acting as a lipid matrix, sebum enriched with antioxidants such as vitamin E and squalene may facilitate the scavenging of ROS and the absorption of ultraviolet radiation, indirectly reducing oxidative and inflammatory stress on keratinocytes and melanocytes [5,41-43].

After characterizing the impact of chemotherapy on the hair follicle and shaft quantitatively by hair loss assessed through the HMI, structurally by shaft alterations ranging from tapering detectable by light and SEM to ultrastructural surface damage, as well as indirectly by changes in melanosome density and patterning as surrogate markers of oxidative stress within the follicular pigmentary unit, these parameters may explain the individual variability in hair retention observed under scalp cooling.

However, further lowering of scalp-cooling temperatures has not improved efficacy in preclinical models when combined with highly cytotoxic regimens such as TAC (simultaneously administered docetaxel, doxorubicin, and cyclophosphamide), underscoring the need for additional protective or restorative strategies [44,45]. Consequently, recent efforts have shifted toward dermatological approaches designed to preserve the follicular pigmentary unit during chemotherapy under scalp cooling, thereby enhancing hair preservation.

Potential strategies to augment scalp-cooling outcomes include topical antioxidants (eg, melatonin and vitamin C encapsulated in nanovesicles) [46]. Another experimental concept involves transient pharmacological cell cycle arrest to reduce chemotherapy susceptibility of follicular keratinocytes. Preclinical studies have demonstrated that topical palbociclib induces temporary G1 arrest in follicular keratinocytes, protecting them from cytotoxic apoptosis while maintaining the subsequent anagen activity [20]. By reducing local perfusion and metabolic activity, scalp cooling limits follicular exposure to cytotoxic agents and lowers chemotherapy-induced oxidative stress. In this context, controlled oxidative stress may paradoxically induce p53-dependent cell cycle arrest rather than apoptosis, consistent with the protective effects of scalp cooling [47], providing a plausible, though not causally established, mechanistic explanation.

Another promising direction involves targeting the transforming growth factor-beta pathway. The chemotherapy-induced upregulation of transforming growth factor-beta 2 promotes catagen transition and apoptosis. The inhibition of this pathway by agents such as cartogenin has shown encouraging results in preclinical models, extending anagen duration and improving follicular resilience. While these findings are promising, clinical validation in human settings is currently lacking. Within this context, the integration of such pharmacological approaches represents a rational translational avenue that warrants further investigation [48,49].

Conceptually, ultrastructural analysis by SEM or TEM provides the most detailed assessment of follicular morphology.

**Figure 4.**
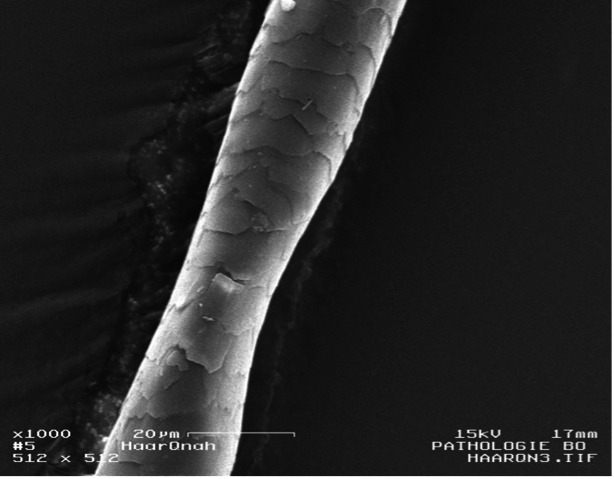
Tapering of hair shaft.

In this study, ultrastructural analyses by SEM and TEM were available for a subset of 52 patients (Figures4  5). This restriction did not reflect a biological or clinical selection criterion but was primarily driven by technical and logistical constraints related to the availability of specialized pathology resources. Consequently, these findings should be interpreted as hypothesis-generating rather than fully representative, while still providing valuable mechanistic insights into follicular structural integrity. However, such techniques are not feasible in routine clinical practice. In everyday settings, macroscopic and light microscopic observations must therefore guide the selection of topical interventions to avoid unnecessary polypharmacy. Light microscopy can reveal surface fragility through cortical irregularities, fissuring, or shaft tapering, features that may reflect oxidative stress as well as impaired matrix activity. Indicators of oxidative stress may further manifest as irregular or diminished pigmentation, uneven melanosome distribution, or translucent cortical segments. Collectively, these structural features may serve as practical surrogate markers of underlying biological vulnerability. However, their predictive validity requires prospective validation before routine clinical applications. Such insights may help stratify patient expectations, inform clinical decision-making regarding scalp cooling, and guide the use of adjunctive restorative strategies to further enhance follicular protection.

**Figure 5.**
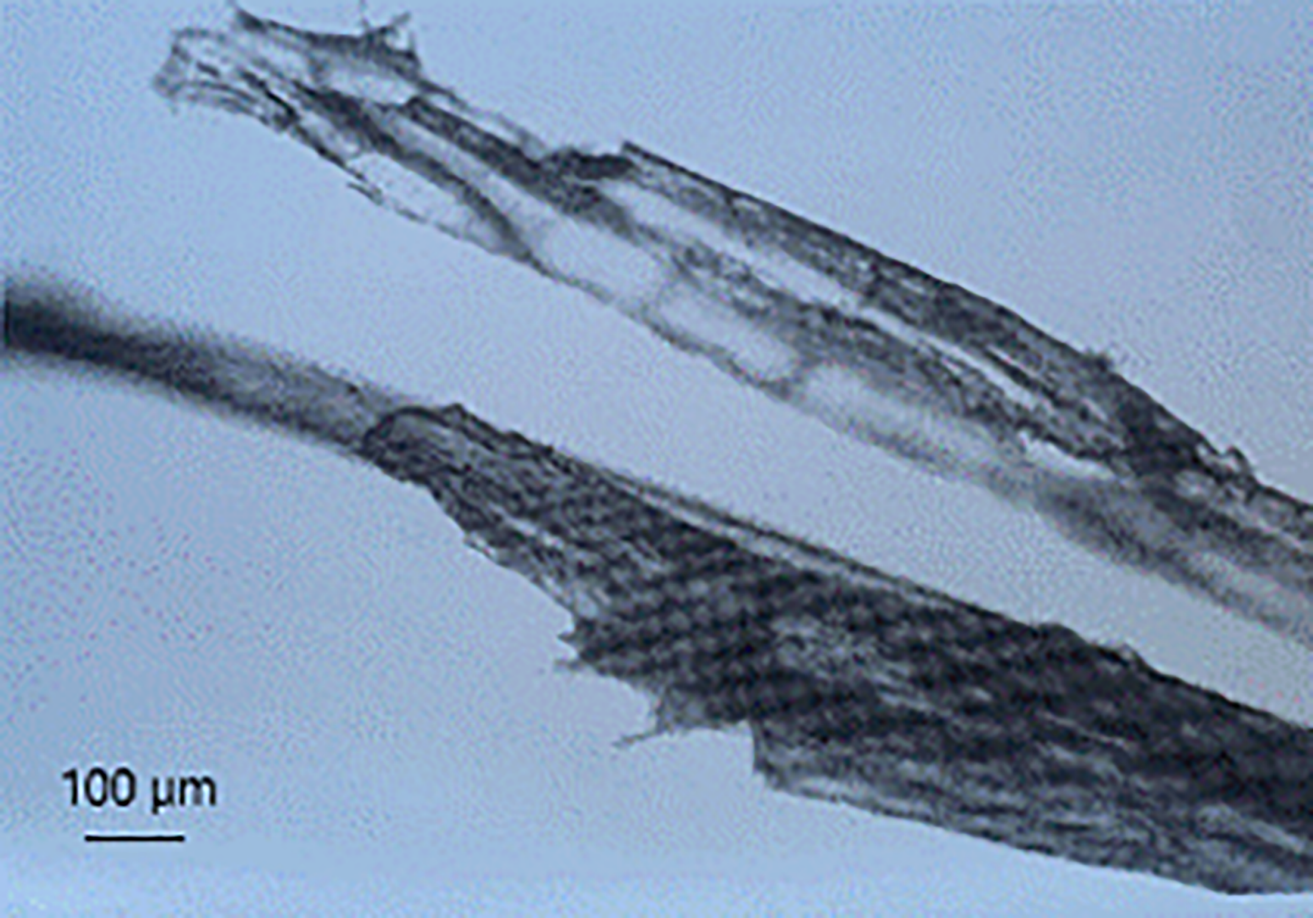
Light microscopic image of the hair shaft showing structural irregularities such as cuticle lifting, focal cuticle disruption, and longitudinal cortical translucencies.

Preventing visible hair loss, defined as an HMI greater than 50 and achieved in 53% of patients, represents not only a biological endpoint but also a meaningful psychological resource, highlighting the broader relevance of scalp cooling beyond esthetics with respect to self-image, quality of life, and potentially treatment engagement. At the same time, the observational single-arm design precludes the causal attribution of psychosocial effects specifically to scalp cooling, as quality-of-life outcomes are influenced by multiple clinical and individual factors.

These observations suggest that patients’ subjective interpretation and personal evaluation of scalp cooling may, in some individuals, be more closely associated with posttreatment quality-of-life measures than the objectively quantified extent of hair preservation itself. However, these relationships should be interpreted cautiously and as associative rather than causally established within the limitations of the present observational design. Accordingly, these findings are best understood as preliminary interpretive observations that require further validation before broader psychosocial conclusions can be drawn. Any potential links between biological hair preservation, ultrastructural follicular findings, and psychosocial outcomes should therefore currently be regarded as descriptive and not as evidence of a directly established mechanistic relationship.

From a psychological perspective, these findings may be interpreted within the framework of cognitive appraisal theory [50]. According to this model, individuals continuously evaluate situations in terms of personal relevance and controllability. Within this context, a positive appraisal of scalp cooling may contribute to a greater sense of security and subjective control during treatment, both of which may act as protective psychological resources. Such resources reduce perceived burden by mitigating feelings of helplessness and uncertainty, while acting as a buffer against stress. In this way, hair preservation under scalp cooling contributes not only to external appearance but also to psychological stability, reinforcing its role as a cornerstone of supportive cancer care [51-53].

In addition, the relative stability of quality of life between preintervention and postintervention assessments may partly reflect the influence of underlying psychological dispositions. However, this pattern is unlikely to be determined by attitudes alone and may also be shaped by patients’ experiences during treatment. These factors are closely linked to body image and psychosocial self-perception, both of which are well-established determinants of quality of life in oncological care [45].

Taken together, these observations suggest that cognitive appraisal represents not only a statistical correlate but also a potentially clinically relevant psychological mechanism. Positive appraisal may be associated with greater resilience and more adaptive coping strategies and could contribute to psychological well-being throughout treatment. Thus, beyond the biomedical effects of scalp cooling, the subjective evaluation of the intervention may represent an important mediator of psychosocial outcomes [50,54-56].

Within the framework of cognitive appraisal, it is conceivable that the integration of adjunctive strategies targeting the follicular pigmentary unit may further support patients’ sense of control and bodily integrity. Interventions aimed at stabilizing pigmentary functions and thereby enhancing the effectiveness of scalp cooling could contribute to a more favorable subjective treatment experience. We hypothesize that patients receiving not only scalp cooling but also adjunctive follicle-directed supportive measures could potentially experience improved coping with the overall burden of cancer therapy. Such an integrative approach may therefore contribute to psychological well-being and maintain quality of life throughout treatment [5,45,57].

The study has several limitations, including its single-center design and the relatively small cohort of 82 patients. The availability of electron microscopy data was limited to the first 52 consecutively enrolled patients. This restriction was determined by the labor-intensive and technically demanding nature of ultrastructural processing and the finite pathology resources available for electron microscopy analyses, rather than by clinical, biological, or outcome-related selection criteria. Consequently, the risk of selection bias within the ultrastructural subgroup is considered low. Although menopausal status was assessed exclusively in female participants, no association with hair preservation was observed in our cohort. Therefore, the absence of an equivalent classification for male participants is unlikely to have substantially influenced the interpretation of our findings and thus represents only a minor limitation.

Consistent with previous reports, no increased risk of scalp metastasis was observed, supporting scalp cooling as a safe option in cancer treatment [58-60].

In summary, this study broadens the current understanding of scalp cooling by identifying the stability of the follicular pigmentary unit and the preservation of shaft integrity as biologically relevant markers of follicular resilience. The findings support a translational perspective in which adjunctive follicle-directed protective strategies may complement scalp cooling. Potential approaches ranging from antioxidant support to the modulation of cell cycle dynamics, for example, by stabilizing or prolonging the anagen phase, remain exploratory and require prospective validation. Collectively, such concepts may contribute to improving the consistency of hair preservation beyond the currently observed response rates of approximately 50% across heterogeneous patient populations. Ultimately, the clinical relevance of these approaches may lie in achieving more reliable treatment-related hair preservation and supporting patient-centered outcomes.

### Conclusion

Taken together, these findings suggest that the clinical relevance of scalp cooling may extend beyond the binary end point of hair preservation. Preserved hair mass was associated with more favorable PROs and appeared to interact with patients’ appraisal of treatment-related changes, supporting a broader biopsychosocial perspective on the treatment experience. While causal relationships cannot be established from this observational study, the results indicate that the impact of scalp cooling may not be fully captured by hair-retention thresholds alone.

By integrating the ultrastructural evidence of follicular vulnerability with patient-reported psychosocial outcomes, this study highlights the multidimensional nature of treatment-related hair loss. These findings support a more nuanced, patient-centered evaluation of scalp cooling and provide a rationale for future strategies aimed at improving both the biological protection of the hair follicle and patients’ experiences during chemotherapy.

## Supplementary material

10.2196/94635 Multimedia Appendix 1 Comprehensive baseline characteristics, treatment-related factors, and posttreatment clinical parameters together with variables derived from light microscopy, transmission electron microscopy, and scanning electron microscopy.

## Abbreviations

CIA: chemotherapy-induced alopecia
EORTC: European Organisation for Research and Treatment of Cancer
HMI: hair mass index
OR: odds ratio
PRO: patient-reported outcome
ROS: reactive oxygen species
SEM: scanning electron microscopy
TAC: simultaneously administered docetaxel, doxorubicin, and cyclophosphamide
TEM: transmission electron microscopy
