# Supplementary material for: Scalp Cooling as a Biopsychosocial Intervention in Patients Receiving Highly Alopecia-Inducing Chemotherapy: Prospective Single-Arm Study
Source: JMIR Cancer. 2026 Jul 20;12:e94635. doi: 10.2196/94635 (PMC13384425; doi:10.2196/94635)
Supplement: Multimedia Appendix 1 [file cancer-v12-e94635-s001.docx]

Supplement

**Table S1**

*Baseline and post-treatment summary*

| Variables | Median (IQR) / Frequency (%) | Variables | Median (IQR) / Frequency (%) |
| --- | --- | --- | --- |
| **Demographics / Baseline** |  | **Post-Treatment** |  |
| Age | 56.5 (49.0–64.8) | HMI | 51.0 (17.0–58.0) |
| Neutrophil nadir | 0.9 (0.5–1.3) | Total Melanosomes | 10.9 (4.6–17.0) |
| Sex=female | 80 (97.6%) | Pheomelanosomes | 5.2 (2.9–12.7) |
| Comorbidity | 31 (38.3%) | Eumelanosomes | 3.8 (1.7–6.0) |
| Medication | 53 (65.4%) | Eu-to-Pheo-ratio | 0.52 (0.30–0.86) |
| Nicotine abuse | 12 (15.0%) | Total Melano Diff | -12.9 (-19.0 – -2.4) |
| Menopause | 56 (75.7%) | Pheomelano Diff | -6.8 (-16.4 – -1.8) |
| Hair length >5cm | 47 (63.5%) | Eumelano Diff | -2.3 (-6.2–0.0) |
| Head cover | 16 (21.6%) | Hair shaft diameter | 59.2 (51.5–69.2) |
| Hair treatment |  | Follicle diameter | 118.5 (92.2–141.4) |
| none | 32 (43.8%) | Hair shaft Diff | -1.8 (-13.1–3.8) |
| thermal | 3 (4.1%) | Follicle difference | -14.0 (-51.0–5.0) |
| chemical | 8 (11.0%) | Bulb size | 121.5 (96.5–138.8) |
| both | 30 (41.1%) | Hair shaft | 62.0 (50.0–74.0) |
| Hair color |  | Anagen bulb | 50.0 (30.0–60.0) |
| gray | 37 (50.0%) | HMI difference | -27.0 (-45.0– -10.0) |
| blond | 10 (13.5%) | Burden Cooling | 2.0 (1.0–4.0) |
| brown | 22 (29.7%) | Appraisal Cooling | 6.0 (5.0–7.0) |
| red | 1 (1.4%) | Quality of life | 5.0 (4.0–6.0) |
| black | 4 (5.4%) | Headache | 2.0 (1.0–3.0) |
| Hair texture |  | Feeling cold | 3.0 (1.0–4.0) |
| straight | 49 (66.2%) | Heaviness | 2.0 (1.0–2.2) |
| wavy | 14 (18.9%) | Neck pain | 2.0 (1.0–2.0) |
| curly | 11 (14.9%) | Other symptoms | 1.0 (1.0–2.0) |
| **Pre-Treatment** |  | Head cover outside | 14 (23.7%) |
| HMI | 75.0 (58.0–92.0) | Damage grade |  |
| Total Melanosomes | 22.0 (11.7–33.1) | 0 | 2 (4.8%) |
| Pheomelanosomes | 15.0 (7.8–24.6) | 1 | 11 (26.2%) |
| Eumelanosomes | 6.0 (2.1–9.8) | 2 | 17 (40.5%) |
| Eu-to-Pheo-ratio | 0.34 (0.21–0.61) | 3 | 12 (28.6%) |
| Hair shaft diameter | 64.8 (58.0–75.2) | Damage grade Diff |  |
| Follicle diameter | 146.5 (110.5–171.9) | -1 | 1 (2.4%) |
| Bulb size | 133.0 (114.5–159.0) | 0 | 8 (19.0%) |
| Hair shaft | 68.0 (58.5–79.5) | 1 | 21 (50.0%) |
| Anagen bulb | 70.0 (60.0–80.0) | 2 | 11 (26.2%) |
| Quality of life | 6.0 (4.0–6.0) | 3 | 1 (2.4%) |
| Damage grade |  |  |  |
| 0 | 16 (30.8%) |  |  |
| 1 | 26 (50.0%) |  |  |
| 2 | 9 (17.3%) |  |  |
| 3 | 1 (1.9%) |  |  |

*Note*. Percentages can differ due to varying number of missing values.

*Demographic and baseline clinical characteristics*

All analyses incorporated the same set of clinically relevant variables previously identified as potential determinants of hair retention: age, menopausal status, comorbidity, medication, nicotine abuse, neutrophil nadir, and prior hair treatment (none vs. rest). Continuous outcomes (HMI post and HMI difference) were analyzed using linear regression models, reported as standardized regression coefficients (β), 95% CI and corresponding p-values. Model fit was expressed as adjusted *R*². The binary outcome (HMI post ≥50) was analyzed using binary-logistic regression. Results are presented as odds ratios (OR) with 95% confidence intervals (CI). Model performance was quantified using Nagelkerke’s *R*².

**Table S2**

*Linear regression on post-HMI (left), logistic regression on visible hair retention (HMI≥50; middle) and linear regression on HMI difference (right) for demographic and clinical characteristics*

| Variables | β | *95% CI* | *p* | *OR* | *95% CI* | *p* | β | *95% CI* | *p* |
| --- | --- | --- | --- | --- | --- | --- | --- | --- | --- |
| Age | 0.20 | -0.12–0.53 | 0.219 | 1.04 | 0.97–1.11 | 0.274 | 0.41 | 0.10–0.72 | 0.011 |
| Menopause | -0.45 | -1.23–0.32 | 0.248 | 0.56 | 0.09–3.18 | 0.518 | -0.22 | -0.96–0.51 | 0.546 |
| Comorbidity | -0.55 | -1.13–0.02 | 0.060 | 0.27 | 0.07–0.98 | 0.053 | -0.30 | -0.84–0.25 | 0.287 |
| Medication | -0.22 | -0.74–0.30 | 0.404 | 0.57 | 0.17–1.80 | 0.343 | -0.54 | -1.04– -0.05 | 0.033 |
| Nicotine abuse | 0.24 | -0.42–0.91 | 0.468 | 4.01 | 0.84–26.21 | 0.106 | 0.49 | -0.15–1.12 | 0.129 |
| Neutrophil | 0.24 | -0.01–0.48 | 0.054 | 1.97 | 1.07–4.31 | 0.055 | 0.19 | -0.04–0.42 | 0.100 |
| Hair treatment | -0.02 | -0.27–0.22 | 0.856 | 0.55 | 0.18–1.61 | 0.282 | 0.08 | -0.16–0.31 | 0.514 |

*Note*. Model fits: adj. *R*² = 0.06 (left), Nagelkerke *R*² = 0.21 (middle), adj. *R*² = 0.15 (right).

No single parameter robustly explained the post-treatment HMI (Table S2, left) but comorbidity (β = -0.55, *p* = 0.060) and neutrophil nadirs (β = 0.24, *p* = 0.054) showed borderline effects while other variables were not significant, reflecting a low explained variance of adj. *R*² = 0.06. Comorbidity (*OR* = 0.27, *p* = 0.053) and neutrophil nadir (*OR* = 1.97, *p* = 0.055) also showed borderline associations with prevention of visible alopecia (Table S2, middle) but no parameter reached statistical significance. The model fit was low to moderate (Nagelkerke *R*² = 0.21). When examining factors with HMI difference, age (β = 0.41, *p* = 0.011) and medication use (β = -0.54, *p* = 0.033) emerged with significant effects (Table 2, right). Older age was associated with less HMI reduction and medication with increased hair loss. Model fit (adj. *R*² = 0.15) was low to moderate. To further elucidate the biological correlates of HMI dynamics, we performed a detailed morphological assessment of hair follicles.

*Light microscopy*

Relevant factors incorporated into the light microscopy models were hair shaft diameter, bulb size, and anagen frequency. For each parameter, both pre- and post-treatment values were included in the models. When assessing post-HMI (Table S3, left), only post-treatment anagen frequency showed a significant positive effect (β = 0.30, *p* = 0.014), indicating that a higher anagen frequency was associated with greater HMI after treatment. Overall model fit was moderate (adjusted *R*² = 0.32). In analyses evaluating parameters associated with an achieved HMI ≥ 50 (Table S3, center), pre-treatment hair shaft diameter demonstrated a significant positive effect (*OR* = 1.06, *p* = 0.034), whereas pre-treatment bulb size showed a small but significant negative relationsship (*OR* = 0.97, *p* = 0.009). Model performance was good (Nagelkerke’s *R*² = 0.41). Finally, in anlayses evaluating factors related to HMI change (Table S3, right), only pre-treatment bulb size emerged as a significant factor (β = -0.36, *p* = 0.039), with larger baseline bulb size corresponding to worse preservation of HMI. The explained variance for this model was very low (adjusted R² = 0.007).

**Table S3**

*Linear regression on post-HMI (left), logistic regression on visible hair retention (HMI≥50; middle) and linear regression on HMI difference (right) for light microscopy parameters*

| Variables | β | *95%CI* | *p* | *OR* | *95%CI* | *p* | β | *95%CI* | *p* |
| --- | --- | --- | --- | --- | --- | --- | --- | --- | --- |
| Hair shaft pre | 0.13 | -0.12–0.39 | 0.299 | 1.06 | 1.01–1.13 | 0.034 | 0.07 | -0.24–0.37 | 0.671 |
| Hair shaft post | 0.23 | -0.05–0.51 | 0.106 | 1.03 | 0.98–1.09 | 0.288 | 0.18 | -0.15–0.52 | 0.283 |
| Bulb size pre | -0.20 | -0.48–0.08 | 0.152 | 0.97 | 0.94–0.99 | 0.009 | -0.36 | -0.69– -0.02 | 0.039 |
| Bulb size post | 0.16 | -0.12–0.44 | 0.266 | 1.02 | 1.00–1.04 | 0.078 | 0.02 | -0.31–0.36 | 0.893 |
| Anagen pre | 0.19 | -0.05–0.43 | 0.119 | 1.05 | 0.99–1.12 | 0.118 | 0.00 | -0.28–0.29 | 0.975 |
| Anagen post | 0.30 | 0.06–0.54 | 0.014 | 1.03 | 0.99–1.07 | 0.164 | 0.11 | -0.17–0.40 | 0.436 |

*Note*. Model fits: adj. *R*² = 0.32 (left), Nagelkerke *R*² = 0.41 (middle), adj. *R*² = 0.007 (right).

These findings were subsequently refined using scanning and transmission electron microscopy, allowing high-resolution analysis of cuticular surface features, cortical organization, melanosome distribution, and ultrastructural follicular integrity.

*Transmission electron microscopy*

In regression models for transmission electron parameters we only looked at pheomelanosome and eumelanosome differences because given the pronounced inter-individual variability in baseline melanosome density and composition (e.g., light vs. dark hair, ethnic background), absolute melanosome counts are not suitable to capture treatment-induced damage. Baseline melanosome abundance reflects inherent pigmentation biology and may therefore be less suitable as a direct medicator of treatment-related follicular stress response. To overcome this limitation, we conceptualized melanosomes as a dynamic surrogate marker of intrafollicular oxidative stress. Accordingly, we focused on pre–post differences rather than cross-sectional levels. When evaluating post-HMI, no evaluated parameter reached significance but pheomelanosome difference had a borderline positive effect (β = 0.28, *p* = 0.063) – the less melanosomes were lost the higher HMI was after treatment. Overall explained variance was low (adjusted *R*² = 0.04). For HMI ≥ 50 there was no significant factor and overall model performance was low (Table S4, Nagelkerke’s *R*² = 0.06). Because melanosome stability is best reflected by change from baseline measures, concordant analysis of hair mass index difference more precisely captures the biologically relevant treatment effect than absolute post treatment values. Finally, for HMI change, pheomelanosome difference showed a significant positive effect (β = 0.36, *p* = 0.009), indicating that less loss was associated with smaller decline in HMI while eumelanosome difference showed a trend in the same direction but did not reach significance (β = 0.25, *p* = 0.063). Model fit was moderate (adj. *R*² = 0.20).

**Table S4**

*Linear regression on post-HMI (left), logistic regression on visible hair retention (HMI≥50; middle) and linear regression on HMI difference (right) for transmission electron microscopy parameters*

| Variables | β | *95% CI* | *p* | *OR* | *95% CI* | *p* | β | *95% CI* | *p* |
| --- | --- | --- | --- | --- | --- | --- | --- | --- | --- |
| Pheo diff | 0.28 | -0.02–0.57 | 0.063 | 1.05 | 0.99–1.12 | 0.149 | 0.36 | 0.10–0.63 | 0.009 |
| Eu diff | 0.04 | -0.25–0.34 | 0.762 | 0.97 | 0.86–1.09 | 0.669 | 0.25 | -0.01–0.52 | 0.063 |

*Note*. Model fits: adj. *R*² = 0.04 (left), Nagelkerke *R*² = 0.06 (middle), adj. *R*² = 0.20 (right).

*Scanning electron microscopy*

In scanning electron microscopy analyses, change scores (post–pre) of hair shaft damage degree were used to capture within-subject structural alterations while accounting for baseline heterogeneity, as this approach more directly reflects therapy-associated changes. Modeling pre- and post-treatment values separately would have complicated interpretation and partly reflected stable baseline characteristics rather than dynamic treatment-related alterations.

Greater increases in damage degree were significantly associated with lower post-treatment HMI values (Table S5; β = −0.32, p = 0.041), indicating that increasing structural hair shaft damage was linked to less favorable hair preservation outcomes. Explained variance, however, remained low (adjusted R² = 0.08).

For visible hair retention (HMI ≥50), damage degree difference did not reach statistical significance (OR = 0.44, p = 0.071). Model performance was low (Nagelkerke’s R² = 0.11).

Finally, in analyses examining HMI change (HMI diff; post–pre), damage degree was not significantly associated with HMI changes over timr, indicating a stronger decline in HMI values over time (β = −0.22,95% Cl -0.54 to 0.09, p = 0.152). Explained variance remained low (adjusted R² = 0.03).

Spearman correlation analyses revealed no significant associations between melanosome-related transmission electron microscopy measures and scanning electron microscopy damage variables (all p > 0.05; Supplement S1).

**Table S5**

*Linear regression on post-HMI (left), logistic regression on visible hair retention (HMI≥50; middle) and linear regression on HMI difference (right) for scanning electron microscopy parameters*

| Variables | β | *95% CI* | *p* | *OR* | *95% CI* | *p* | β | *95% CI* | *p* |
| --- | --- | --- | --- | --- | --- | --- | --- | --- | --- |
| Damage diff | -0.32 | -0.62– -0.01 | 0.041 | 0.44 | 0.17-1.01 | 0.071 | -0.22 | -0.54-0.09 | 0.152 |

*Note*. Model fits: adj. *R*² = 0.08 (left), Nagelkerke *R*² = 0.11 (middle), adj. *R*² = 0.03 (right).

**Table S6**

*Descriptives of post-HMI by damage grade post*

| Damage grade | n | Mdn | Q1 | Q3 |
| --- | --- | --- | --- | --- |
| 0+1 | 13 | 57.0 | 52.0 | 72 |
| 2 | 17 | 54.0 | 46.0 | 68 |
| 3 | 12 | 24.5 | 12.5 | 51 |
